# Validity must be discussed

**DOI:** 10.1080/02813432.2019.1641901

**Published:** 2019-07-16

**Authors:** Karin Starzmann

**Affiliations:** R&D Centre Skaraborg Primary Care, Skövde, Sweden;

I have some concerns about the article “Unsanctioned techniques for having sickness certificates accepted: a qualitative exploration and description of the strategies used by Swedish general practitioners” written by Mani Shutzberg [1]. At first the informants are described as GP:s in Sweden. I believe that Dr Shutzberg have a mix of GP, GP-trainee and interns in the study. It is impossible to be a GP in Sweden, at 29 of age and only have 0.5 years of experience from primary care. The experience of a physician is of most importance in how a physician handle different problems.

The second concern is about that the study only have one author. How is it possible to triangulate the result? He has some supervisors, but he doesn’t let us know who they are. In a qualitative study are the context were the study is conducted of most interest.

The third concern is the interview guide were the informants are asked in the first question *“Which aspects of the sickness certification process do you find most difficult? Examples?”* This makes this study biased as a qualitative exploring study from the beginning. By saying “most difficult” he influences the informants to describe a certain part of their work with sick leave certifications. Maybe can this be used in a quantitative study but not in a qualitative.

The fourth concern is about the DFA-chain. Dr Shutzberg writs that the DFA-chain is introduced in 2009, this is wrong, it was introduced already in 2005. He doesn’t describe how the WHO classification ICF (International classification of functioning) are linked to the DFA-chain [2]. It seems that he doesn’t know. This is a qualitative study were the author doesn’t know the most fundamental theories about how the Swedish sick leave certificate are constructed. In his references ICF is described [3]. Has he read the references? The result from the interviews must be compared to the theory of ICF. He doesn’t mention activity limitations. He uses the word work ability in the discussion instead of activity limitations. In the Swedish certificate activity limitations must be described, not work ability. It is possible that both the physicians in the study and the author don’t know what the Social insurance officer are expecting in the certificates. If this is the fact, then the result shows their lacking skills in how to formulate a sickness certificate and the result are a description of mental defends. If they don’t know how to formulate a sickness certificate out of Swedish standards, it is an easy way out to use different technics to get the patient certified sick.

Out of this do I question the validity of the study. To describe the informants in the wrong way is a serious mistake and to exclude fundamental theories diminish the result of the study.
