# Supplementary material for: Validity must be discussed
Source: Scand J Prim Health Care. 2019 Jul 16;37(3):388–9. doi: 10.1080/02813432.2019.1641901 (PMC6713171; doi:10.1080/02813432.2019.1641901)
Supplement: Related texts

# Author’s reply

**DOI:** 10.1080/02813432.2019.1641901

**Authors:** Mani Schutzberg

**Affiliations:** Centrum för praktisk kunskap (Center for practical knowledge), Södertörn University, Huddinge, Sweden

The findings of my article published in SJPHC [1], which showed the different informal and unsanctioned ways in which primary care physicians can navigate in the sickness certification process, seem to displease Dr. Starzmann. The preferred course of action is therefore to question its validity. I will address her concerns in the order presented in her letter to the editor.

1. As Starzmann points out, the experience of the primary care physician informants in my study varies. A deliberate decision was made to include specialists, trainees and interns, to ensure that the informants reflect the “demographics” of those who actually work at primary health clinics. Starzmann takes issue with the term used to designate this group, claiming that the term GP should only be reserved for specialists. I have found no such definition for the English term “GP,” but it is true that a customary distinction is made between specialists and “primary care physicians” in the literature. However, I fail to see how it puts the validity at serious risk. The aim of the study was not to compare how different groups of physicians use techniques, but rather to explore and describe the different techniques deployed. Including only specialists would be more problematic in this regard.

2. The reason for having one author is stated in the acknowledgement section of the article. The supervisors—professor in the theory of practical knowledge Fredrik Svenaeus and associate professor in the theory of practical knowledge Jonna Lappalainen—have participated in the analysis of the collected data as described. However, none of them qualify as co-authors according to the slightly narrower publication tradition of our humanistic field.

3. I am perplexed by Dr. Starzmann’s third concern, that a qualitative study aiming to explore a difficult aspect of medical practice skews the data by asking about that very same difficult aspect of medical practice. The aim of the study is clearly stated in the introduction of the article. Interview studies present many interesting and difficult challenges to validity, some of which I have addressed in the article, and will expand upon in my dissertation. The concern raised by Starzmann about the phrasing in one of the interview questions in the interview guide is, however, not the most urgent.

4. The fourth concern—also the one most passionately expressed—focuses on the lack of theoretical understanding of concepts. Allegedly, both GPs and I succumb to this ignorance of the inner workings of the DFA-chain and its conceptual genealogy that traces back to the ICF. Dr. Starzmann seems to believe that if only GPs could be made to truly understand the concepts, there would not be a problem, no friction between GPs and the Swedish Social Insurance Agency (SSIA). By elevating to this level of abstraction, Starzmann misses what the article actually aims for: “to explore the informal and unsanctioned techniques employed by GPs as a means to increase the likelihood of sickness certificate approval”.

I believe that Starzmann’s position reveals a deeper flaw in established thought concerning relations between institutions and its grassroots. After the 1953 popular uprising in East Germany, the poet Bertolt Brecht remarked that the East German state preferred “to dissolve the people and elect another”, rather than to criticize itself [2, p. 119]. When ideals and reality clash, too often reality is forced to submit, or declared invalid. I think Brecht’s insight carries over to the relationship between the SSIA and GPs, as well as to Starzmann’s reproach.

## References

[1] ShutzbergM Unsanctioned techniques for having sickness certificates accepted: a qualitative exploration and description of the strategies used by Swedish general practitioners. Scand J Prim Health Care. 2019;37(1):10–17.10.1080/02813432.2019.1569426PMC645441030689481

[2] BrechtB, GrimmR Poetry and prose. New York (NY): Continuum; 2003.
